# Binary Amplitude Reflection Gratings for X-ray Shearing and Hartmann Wavefront Sensors

**DOI:** 10.3390/s21020536

**Published:** 2021-01-13

**Authors:** Kenneth A. Goldberg, Antoine Wojdyla, Diane Bryant

**Affiliations:** Advanced Light Source, Lawrence Berkeley National Laboratory, Berkeley, CA 94720, USA; AWojdyla@lbl.gov (A.W.); DBryant@lbl.gov (D.B.)

**Keywords:** X-ray, wavefront, shearing, Hartmann, Talbot, grating, interferometry, aberrations

## Abstract

New, high-coherent-flux X-ray beamlines at synchrotron and free-electron laser light sources rely on wavefront sensors to achieve and maintain optimal alignment under dynamic operating conditions. This includes feedback to adaptive X-ray optics. We describe the design and modeling of a new class of binary-amplitude reflective gratings for shearing interferometry and Hartmann wavefront sensing. Compact arrays of deeply etched gratings illuminated at glancing incidence can withstand higher power densities than transmission membranes and can be designed to operate across a broad range of photon energies with a fixed grating-to-detector distance. Coherent wave-propagation is used to study the energy bandwidth of individual elements in an array and to set the design parameters. We observe that shearing operates well over a ±10% bandwidth, while Hartmann can be extended to ±30% or more, in our configuration. We apply this methodology to the design of a wavefront sensor for a soft X-ray beamline operating from 230 eV to 1400 eV and model shearing and Hartmann tests in the presence of varying wavefront aberration types and magnitudes.

## 1. Introduction

Recent advances in high-coherent-flux X-ray light sources have spurred the development of beamline optical systems that can preserve the wavefront quality of focused beams. Scientific applications of synchrotron and free-electron laser light, such as ptychography [1] and X-ray photon correlation spectroscopy [2], rely on well-controlled, stable, coherent beams. In this endeavor, in situ wavefront sensing feedback performed at the operational wavelengths is central to the feedback required to achieve and maintain optimal performance.

As with any optical system, wavefront quality is measured in fractions of a wavelength. When the wavelengths are nanometers or angstroms, the challenges associated with the accurate wavefront sensing increase. The past three decades have seen numerous successful demonstrations, in a variety of circumstances. Among these, shearing interferometry [3,4,5,6,7,8] and Hartmann wavefront sensing [9,10,11,12] (referred to as shearing and Hartmann) have emerged as rapid, high-sensitivity methods for measuring the wavefront slope and shape and for feedback to the shape of bendable mirrors. These techniques are relatively simple to implement and easy to align and use, and the data can be recorded in a single exposure and interpreted quickly. Widely used with visible-light, the Shack–Hartmann technique has also been demonstrated at a 46.9 nm wavelength, using diffractive lenslets in reflection [13].

**Figure 1:**
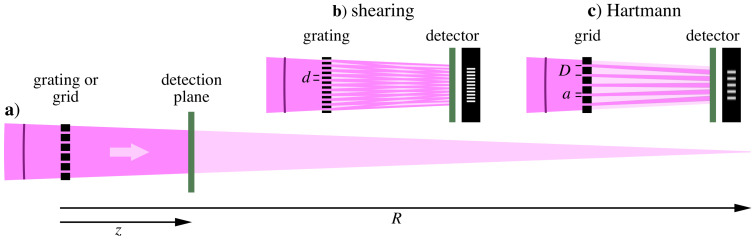
(**a**) Beam geometry used in the single-grating shearing interferometry and Hartmann wavefront. A converging (shown) or diverging beam, incident from the left, is transmitted by a shearing grating or Hartmann grid and propagates a distance *z* to the detection plane. The grating-to-focus distance is *R* (negative in the diverging-beam case). Inset details show (**b**) shearing geometry with grating pitch *d* and (**c**) Hartmann geometry with grid pitch *D* and opening width *a*.

Shearing and Hartmann share similar geometries and compatible hardware requirements, yet they operate on different principles (Figure 1). Shearing is an interferometric technique that uses a 1D or 2D grating structure to generate multiple, laterally displaced, and overlapping copies of the wavefront at a downstream detection plane. Hartmann is a non-interferometric approach that commonly uses a grid of holes in an opaque screen to project an array of isolated spots (2D) or lines (1D) onto the detector. Both techniques are sensitive to the wavefront slope. Between these two complementary approaches, Hartmann has greater capacity to measure large aberrations, while shearing offers higher sensitivity for small aberrations and a higher measurement-point density.

By using the terms grating and grid for shearing and Hartmann elements, respectively, we wish to draw a distinction between (a) the interference properties inherent in shearing and (b) the spot-projection mode of Hartmann.

### New Challenges

At-wavelength metrology for new, high-coherent-flux X-ray beamlines introduces complications that require innovation. Our present motivation is the design of wavefront sensors for direct feedback to adaptive mirrors on four insertion-device beamlines in the Advanced Light Source Upgrade project (ALS-U). We present a solution for adaptive optic feedback in the presence of one-dimensional beam dispersion (upstream of a grating monochromator’s exit slit), narrow beam widths, and high power.

On these beamlines, a varied-line-spacing (VLS) planar grating monochromator performs vertical focusing and spectral dispersion [14], while in the horizontal direction, a plane-elliptical adaptive mirror focuses the beam and corrects wavefront aberrations. Horizontal and vertical elements separately focus to the exit slit plane of the monochromator. Upstream of the exit slit, the converging beam contains the combined aberrations from all elements, yet dispersion spreads the beam vertically, frustrating direct measurement of the wavefront in that direction. The adaptive mirror performs shape correction in the horizontal direction only, so one-dimensional wavefront sensing feedback is sufficient for it. Vertical wavefront sensing and adjustment are performed separately, using the monochromator’s mirror and grating angles together, for fine focusing.

Wavefront sensors must accommodate beamlines operating across broad photon-energy ranges. We previously demonstrated the concept of a compact array of simultaneously-illuminated, one-dimensional gratings on a membrane, in a transmission geometry [7]. However, in this application, transmission membranes cannot survive the high power load. The converging beam’s width can vary from several millimeters down to 0.5 mm (±1σ), and it can carry up to 0.5 W of X-ray power. We calculate that the 100 nm thick, absorber-coated membranes commonly used for soft X-ray shearing interferometry would melt with temperature increases of hundreds to thousands of degrees [15]. Therefore, we are developing a new class of dedicated reflection gratings for both shearing and Hartmann. Small, glancing incidence angles stretch the beam footprint, reducing the power density by 1/sinθ, and silicon substrates promote heat dissipation and active cooling, where necessary.

Whereas monochromator gratings (blazed, lamellar, etc.) are patterned mirror elements that typically modify the phase (i.e., path length) of the incoming wave, Hartmann requires high-contrast amplitude modulation. Phase gratings are used for shearing at X-ray wavelengths [8,16], but optimization is challenging to achieve in reflection, over a large photon energy range, at fixed incidence angles. We developed binary, amplitude-modulating reflection gratings to serve both shearing and Hartmann testing with a compact optical element. The gratings are illuminated in an off-plane mounting, conical-diffraction geometry [17]. The creation and metrology of these new elements were described in a previous report [18].

## 2. Shearing Grating and Hartmann Grid Design

The inherent wavelength dependence of the shearing and Hartmann test geometry (described below) raises a fundamental design decision for wavefront sensors that are required to span a spectral range. Either the distance between the grating and the detector must vary or there must be an array of gratings at a fixed distance. Using a fixed detector distance and a compact array of gratings and grids simplifies the mechanical system. Our design allows shearing and Hartmann to use the same grating-to-detector distance, with gratings and grids patterned on the same optical element.

### 2.1. Binary Amplitude Shearing Grating Design

Various approaches to single-grating lateral shearing interferometry have been demonstrated, including many for short-wavelength applications. Among them are the Ronchi test [19], the double-frequency grating [20], and the sideband-filter methods [21,22]. Most relevant to the present work is the carrier-frequency approach of Naulleau et al. [3], for which Siegel provides a physical analysis [23]. Among the earliest short-wavelength demonstrations were Bjorkholm et al. [24] and Ray-Chaudhuri and Krenz [25]. More recent examples include Merthe et al. [6], Wojdyla et al. [7], Seaberg et al. [8], Assoufid et al. [16]. The authors referred to this unfiltered, high-beam-overlap approach as digital Talbot interferometry, single-grating Talbot interferometry, and Ronchi shearing interferometry, although Ronchi’s configuration shears the diffracted beams by half of the width of the pupil.

In the measurement geometry shown schematically in Figure 1, the distance from the grating to focus is *R*, and the distance from the grating to the detector plane is *z*; both *R* and *z* are positive quantities. With diverging beams, R<0. We can take R→±∞ for plane-wave illumination.

Through constructive interference, shearing produces high contrast self-images (called Fresnel images by Winthrop and Worthington [26]) in planes downstream of the grating; these are called Talbot planes for the Talbot self-focusing effect [27,28]. Whether the grating is an amplitude-modulating or phase-modulating element affects the planes in which high-contrast fringes appear.

The distance, *z*, to the first high-contrast Fresnel image occurs where the first diffraction order of the grating has been laterally deflected by one grating period; in this way, it interferes constructively with the undiffracted zeroth order and the symmetric −1st order. The deflection angle is given by the grating equation, sinθ=λ/d. Relative to the grating pitch *d*, the effective grating pitch $d^{\prime }$ observed in the measurement plane is scaled geometrically by the beam magnification (Fresnel scaling theorem [29,30]), whether converging or diverging:

(1) $$d^{\prime }=\frac{d\left(R-z\right)}{R}.$$

The condition for the first Fresnel image is thus $z\sin\theta =d^{\prime }$, and it may be written as:

(2) $$\frac{z\lambda }{d}=\frac{d(R-z)}{R}.$$

Solving for *z* yields:

(3) $$z=\frac{d^{2}R}{\lambda R+d^{2}}.$$

As R→±∞, $z\to d^{2}/\lambda$. We note that this is half of the conventional Talbot distance derived by Rayleigh for plane waves [28].

This same approach identifies planes where beam overlaps are multiples of the effective grating pitch, $nd^{\prime }$. This yields a more general form of Equation (3), describing a series of Talbot planes.

(4) $$z_{n}=\frac{nd^{2}R}{\lambda R+nd^{2}}.$$

In many applications, λR is much larger than $d^{2}$, and the Talbot planes are almost evenly spaced in *z*. Additional planes of relatively high intensity contrast occur at certain fractional Talbot distances [31], which are not essential to our application.

With a fixed grating-to-detector distance, we design an array of gratings, individually tuned for specific photon wavelengths and usable across a range, discussed below. Designing for the first diffraction order and the first Talbot plane, we can define an effective distance [29],

(5) $$z^{\prime }=\frac{zR}{R-z}.$$

Then, for each wavelength, we have a corresponding pitch:

(6) $$d=\sqrt{\frac{\lambda zR}{R-z}}\;=\sqrt{\lambda z^{\prime }}\;.$$

Equivalently, we can write this in terms of the photon energy, *E*, substituting λ=hc/E. hc is the product of the Plank constant and the speed of light in a vacuum, equal to 1239.842 nm/eV.

From Equation (4), given *z*, *R*, and *d*, the set of wavelengths that satisfy the Talbot condition may be written with either *z* or $z^{\prime }$:

(7) $$\lambda_{n}=\frac{nd^{2}}{z}\frac{\left(R-z\right)}{R}=\frac{nd^{2}}{z^{\prime }}.$$

In the reconstruction of the wavefront, or its slope, an initial phase demodulation step treats the measured signal as being proportional to a discrete derivative of the wavefront, in the direction of the shear. The physical scaling factor is called the shear magnitude, and it is equal to the lateral displacement of the beam. In the first Talbot plane, the shear magnitude is $d^{\prime }$; in other Talbot planes, it scales in proportion to *z*. The shear magnitude can be used to increase the sensitivity of the measurement, at the expense of the spatial frequency response [23].

### 2.2. Hartmann Wavefront Sensor Grid Design

The Hartmann test probes the wavefront slope. Diffraction spreads the beamlets as they propagate, with the local slope shifting the beamlet positions laterally. The measured positions and known distance to the detector plane reveal the slope or slope error. In transmission geometry, the open areas are holes in an absorbing screen. In reflection geometry, the reflective lines serve the same role; therefore, we describe them in terms of line widths.

High-precision beamlet position measurement requires lateral separation, and it is helpful to have smooth intensity profiles. The line width, *a*, governs the diffracted width; the pitch, *D*, determines the lateral spacing. These parameters couple with the wavelength and geometric parameters in the design optimization. Following Equation (1), the pitch scales geometrically from *D* to *D*′ in the measurement plane. Measurement sensitivity depends on the grid-to-detector distance and the measured beamlet widths: too narrow, and the precision is limited by the resolution of the detector; too wide, and the sensitivity to small shifts is reduced [32]. Furthermore, when line widths are too large, the recorded image becomes a projected shadow of the grid, and the sensitivity is lost. Similar to the shearing case, the wavelength dependence of the design requires that we create an array of grids to span an operating range.

Hartmann grid design begins by setting the line width, which we can select empirically. Slit- or line-diffraction behavior is well described by the Fresnel number [33], $N_{F}=a^{2}/4\lambda z$, for line-width *a* and plane-wave illumination. With a converging or diverging beam, *z* is replaced by $z^{\prime }$ (Equation (5)).

Calculations of the measured beam intensity are made with scalar diffraction theory, applying the Huygens–Fresnel principle [33]. We approximate the gratings and grids as ideal, opaque screens with features much larger than the wavelength. In all cases, we use spatially coherent, monochromatic, uniform-amplitude beams with phase profiles that represent a plane or a converging wave.

**Figure 2:**
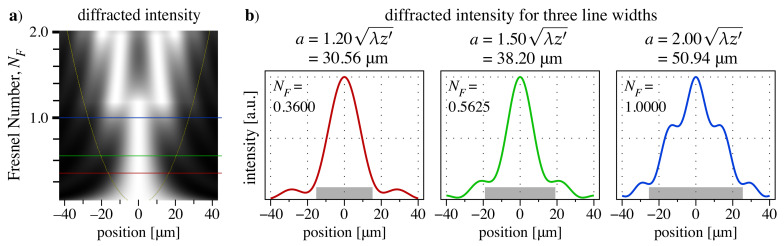
Diffracted intensity patterns from a range of isolated Hartmann-grid line widths calculated with respect to the Fresnel number, $N_{F}=a^{2}/4\lambda z^{\prime }$, with fixed wavelength, λ=2nm, detector distance, z=0.3m, and incident beam radius, R=4m. Note that $\sqrt{\lambda z^{\prime }}=25.469\mu m$. (**a**) Each row is calculated independently and normalized to its maximum value. Yellow curves show the line width, references to the horizontal axis. Red, green, and blue lines show the positions of three specific diffracted intensity calculations (**b**) extracted from the plot. Gray rectangles represent the line width in each case.

Calculations done in this manner, of the single-line diffraction pattern as a function of the line width, are shown in Figure 2. We selected a line width of:

(8) $$a=1.50\sqrt{\lambda z^{\prime }}=1.50\sqrt{\frac{\lambda zR}{R-z}\;,}$$

with Fresnel number $N_{F}=0.5625$ as our design rule.

**Figure 3:**
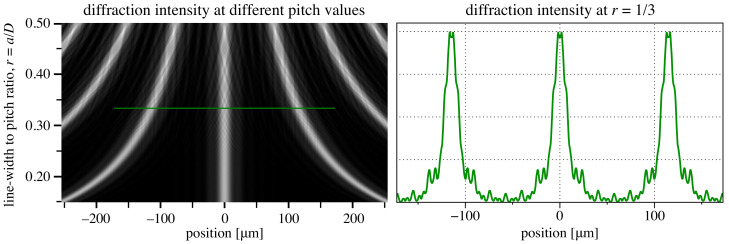
Coherent wave-propagation calculations of Hartmann grid diffraction intensity patterns as a function of the pitch, *D*. A constant line width, a=38.203 μm, is maintained (Equation (8)). The plot on the right shows three periods of the pattern, calculated at r=1/3, extracted at the position of the green line.

The grid pitch *D* can scale with *a* using the duty cycle ratio, *r*, defined as r=a/D, where 0<r<0.5. The optimization of *r* minimizes beamlet overlap while preserving a sufficient number of measurement points across the incident beam. Figure 3 shows the diffraction intensity patterns for a range of *r* values and the fixed *a* value, defined above. We select r=1/3 as a compromise between beamlet isolation and measurement-point density. Substituting Equation (8), the pitch is:

(9) $$D=\frac{a}{r}=3a=4.50\sqrt{\frac{\lambda zR}{R-z}}\;.$$

The Hartmann pitch is 4.50 times larger than the shearing’s (Equation (6)). Since the Talbot distance scales approximately as pitch-squared (Equation (4)), we note that the first Talbot plane in the Hartmann configuration would occur at $20.25\;(4.5^{2})$ times farther downstream than the equivalent shearing case.

The design parameters reached in Equations (8) and (9) emerge from the compromises discussed above and satisfy our design requirements. Other applications may choose different line widths or spacings in order to, for example, operate over larger or narrower wavelength ranges or with different detector distances.

## 3. Shearing and Hartmann Usable Bandwidth and Array Creation

Each shearing grating and Hartmann grid operates across a range of photon energies, and arrays of gratings and grids enable us to span a wide, continuous operating range. Coherent wave-propagation models reveal the useful energy range of individual gratings and grids, enabling us to calculate the energy spacing required in the design and how many gratings are required for a given application.

### 3.1. Number of Gratings Required to Span a Range

We observe that each grating or grid operates across an energy range proportional to its central design energy, *E*, that is within E(1±β). To cover the range in a piecewise-continuous manner, the next central energy in the series follows a geometric progression. Individual gratings designed for energy values $\left\{E_{0},E_{1},\ldots ,E_{N-1}\right\}$ can be spaced according to:

(10) $$E_{j}=E_{0}{\left(1+\beta \right)}^{j},$$

with integer *j* counting from zero. Following this progression, each grating covers a range around $E_{j}$, from Ej(1+β)−12 to Ej(1+β)12. For small β values, this is approximately $E_{j}\left(1-\beta /2\right)$ to $E_{j}\left(1+\beta /2\right)$. To span an energy range from $E_{\min}$ to $E_{\max}$ using *N* gratings requires:

(11) β=(Emax/Emin)1N−1,

with gratings centered at energies Ej=Emin(1+β)j+12 and j={0,1,…,N−1}.

If instead a design requires spacing in energy by relative steps of 1+β, then the number of gratings needed to span a range is an integer, *N*, such that:

(12) $$N\geq \frac{\ln\left(E_{\max}/E_{\min}\right)}{\ln\left(1+\beta \right)}.$$

### 3.2. Usable Bandwidth Considerations for Shearing and Hartmann

Coherent wave-propagation calculations for binary-amplitude shearing and Hartmann methods reveal the practical spectral bandwidth ranges of gratings and grids.

**Figure 4:**
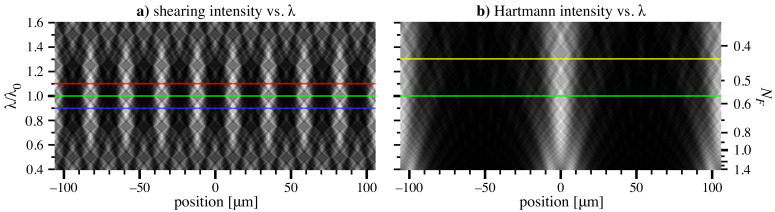
Coherent wave-propagation models of shearing and Hartmann detector-plane intensities for a range of wavelengths about $\lambda_{0}$ = 2 nm (green line), in a converging beam. Each row is normalized for constant integrated power. Pitch values are d=25.469 μm for shearing and D=114.609 μm for Hartmann with a=38.203 μm. (**a**) Red and blue reference lines represent a ±10% wavelength change. (**b**) Hartmann calculations show separation between neighboring beamlets. The yellow line marks a +30% wavelength change, where β = 0.690. The corresponding Fresnel number is shown at the right.

For shearing interferometry, Figure 4a shows calculations for a one-dimensional grating, performed for a fixed grating-to-detector distance of z=300 mm, an ideal, converging cylindrical beam focused to R=4 m from the grating, and a central, soft X-ray wavelength of $\lambda_{0}=2\;nm$. The grating pitch is d=25.469 μm (Equation (6)). One-dimensional intensity patterns are shown for a range of wavelengths about the central energy. High contrast and a strong fundamental spatial frequency are achieved in a range near the central wavelength, with a reproduction of the incident square-wave intensity pattern occurring at the center. Fractional Talbot positions reproduce the pattern with reduced contrast at wavelengths of $0.75\lambda_{0}$ and $1.25\lambda_{0}$. The pattern contrast has local minima with $0.50\lambda_{0}$ and $1.50\lambda_{0}$.

Qualitatively, we observe that a safe selection of β is ∼0.2, meaning that a wavelength range of approximately ±10% can be apportioned to each grating.

Hartmann calculations were made in the same measurement geometry, shown in Figure 4b. The grid pitch D=114.609 μm and line width a=38.203 μm (r=1/3) were calculated from Equation (9). Beam overlapping depends sensitively on *D* and *a*, but only becomes significant for wavelengths longer than the central design wavelength.

Among other factors, the useful operating range of a single grid depends on the data analysis method that will be applied, which determines how well the beamlets must be isolated to achieve a target precision. In this case, it appears that the design can accommodate a ∼30% increase of wavelength without significant overlapping. This corresponds to β=0.6900.

### 3.3. Shearing Bandwidth in the Talbot Planes

**Figure 5:**
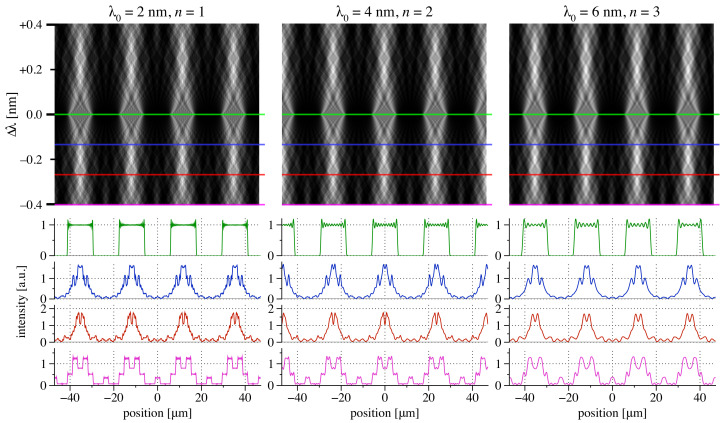
Coherent wave-propagation calculations of the detector-plane, central intensity region in shearing interferometry, for a 2, 4, and 6 nm wavelength, with a fixed geometry matching Figure 4a. Eighty, uniformly-illuminated grating lines are modeled in each case. Conditions match the first three Talbot planes, respectively. Each row is normalized for constant integrated power. Green lines indicate the Talbot condition. Blue, red, and magenta lines show wavelength shifts of −0.133 nm, −0.267 nm, and −0.400 nm, respectively. Corresponding intensity profiles are extracted from the wavelength offsets and plotted below.

Accessing multiple Talbot planes provides a potential way to use a single grating at different wavelength ranges. In the fixed shearing geometry from Figure 4a, we study the intensity patterns near the Talbot condition, for Talbot planes n=1, 2, and 3. Following Equation (7), the Talbot planes are evenly spaced in wavelength increments (e.g., 2, 4, 6 nm). As shown in Figure 5, similar intensity patterns are replicated at these three central wavelengths with respect to absolute (not relative) changes in wavelength. The implications of this are that relative to the central wavelengths, a given useful operating wavelength range about $\lambda_{0}$ (*n* = 1) drops to one-half for $2\lambda_{0}$ (*n* = 2) and to one-third for $3\lambda_{0}$ (*n* = 3). This condition can be useful, but it would require a larger number of gratings to build an energy-range spanning array from the higher Talbot planes.

## 4. Shearing and Hartmann Design Example

Among the new soft X-ray beamlines being developed for the upgrade of the Advanced Light Source, one operates across the energy range 230 eV to 1400 eV. At the position of the 1D wavefront sensor, upstream of the exit slit, the illuminating beam is as small as 1.5 mm wide (±3σ) and converges to focus with an *R* = 4 m radius. A grating-to-detector distance of 0.3 m was selected as a compromise between Hartmann and shearing design requirements in this energy range.

**Figure 6:**
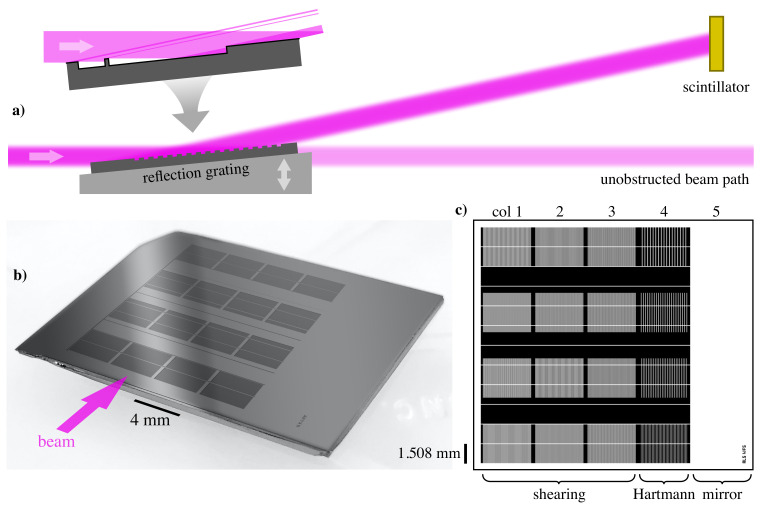
Illustration of the wavefront sensor grating array, its pattern, and use. (**a**) The narrow beam has a 2°, glancing angle of incidence and a longitudinal footprint longer than the array, so the four patterns in a column are illuminated at once. The inset grating cross-section detail shows how light reflects from the top surface, yet all other light is blocked by the thin walls and etched pattern features. At low angles, the detection-plane scintillator may be upright or inclined. The pattern (**c**) is etched into a silicon wafer chip (**b**), which is approximately 28 mm wide. The pattern’s five columns contain 12 shearing gratings (Columns 1–3), four Hartmann grids (Column 4), and an open mirror region (column 5).

Here, we calculate the properties of the gratings and grids required to span this range and then show how they will be arranged in a reflective array design. Figure 6a shows the measurement geometry, with a rapidly insertable, glancing-incidence grating array deflecting the beam upward toward the scintillator—typically a polished, cerium-doped, yttrium aluminum garnet (YAG) crystal. A visible light microscope magnifies the interference pattern and records the images for processing.

### 4.1. Shearing Grating and Hartmann Grid Arrays

**Figure 7:**
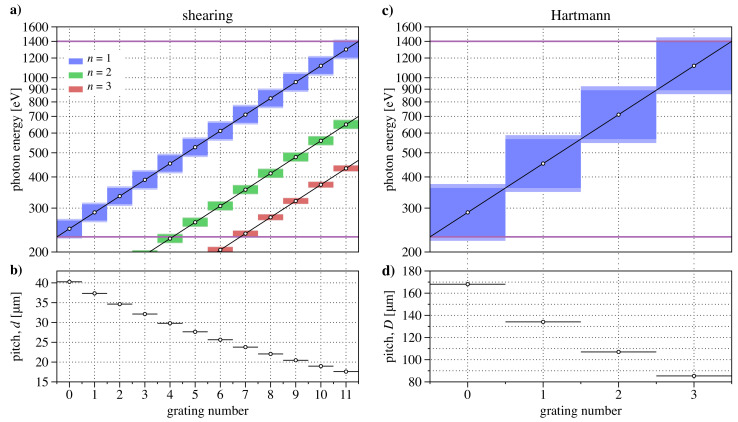
Design of shearing and Hartmann arrays spanning a 230 eV to 1400 eV energy range. (**a**) Counting 0 to 11, twelve gratings span the range with β=0.1624, using the first Talbot plane (n=1). Blue-colored regions show the individual gratings’ operating energy range. The equivalent energy range is shown for the second and third Talbot planes in green and red. (**b**) Corresponding grating pitch values. (**c**) With β=0.4351, four Hartmann grids (Numbered 0 to 3) span the range. (**d**) Corresponding Hartmann grid pitch values.

Our array design has space for 12 shearing gratings arranged in Columns 1, 2, and 3, as shown in Figure 6c. Equation (11) shows that with N=12, energy spacing with β=0.1624 will span the range. In practice, each grating can be used over a slightly larger range of energies. Figure 7a shows the central energy values and operating ranges for the 12 gratings. The corresponding grating pitch values, calculated from Equation (6), are shown in Figure 7b. The energies and ranges are also plotted for second and third Talbot plane conditions from these same gratings.

Similarly, for a Hartmann array with N=4 grids, β = 0.5707. The central energy values and ranges are shown in Figure 7c. The corresponding grid pitch values, *D*, are calculated from Equation (9) with r=1/3 and are shown in Figure 7d.

### 4.2. The Etched Grating Chip

As described in [18], the array of one-dimensional patterns is deeply etched (> 15 μm) into a prime-grade silicon wafer and cleaved into chips. An illustration of the grating, designed for a 2° glancing angle of incidence, is shown in Figure 6b. The columns are 4 mm wide, and the individual gratings and grids measure 3.700 mm by 3.013 mm, with a 2.000 mm vertical separation between patterns (separation reduces overlap from the vertical dispersion.)

At this small angle, the projected height of the array (i.e., scaled by sinθ) is 0.630 mm, which is smaller than the beam height in cross-section, so all four gratings in a 18.050 mm column are illuminated at once. The gratings have an equivalent cross-sectional height of 105 μm with a vertical separation of 70 μm. During the measurement, the grating that most closely matches the designed operating energy is selected from the measured intensity pattern for analysis, as described below. Different columns are selected by translating the chip laterally.

A pattern of 50 μm-thick horizontal walls, spaced by 1.508 mm, blocks light reflected from the etched regions from propagating toward the detector. In this way, the gratings produce a binary reflected amplitude and behave much like transmission gratings at normal incidence. Projected onto the beam in cross-section, the walls have an effective vertical width of 1.7 μm. Because of this small size and their uniformity in the horizontal direction, they contribute negligibly to the measured intensity and do not affect the analysis of the intensity modulation in the horizontal direction.

## 5. Modeling

Coherent wave-propagation modeling calculates the detector-plane intensity patterns under different conditions, demonstrating the operation of the device. The following examples show the predicted appearance of shearing and Hartmann measurements with an ideal incident beam and then with the addition of various aberrations using the geometry described above.

Dispersion spreads the beam in the vertical direction, perpendicular to the wavefront measurement. In this example, the monochromator grating is 5.5 m from the focus. The beamline achieves a spectral resolution of 5000 with a 15 μm vertical exit slit. With an inherent bandwidth of 1% FWHM, this leads to an angular spread of 136 μrad, illuminating the wavefront sensor chip. Thus, points on the grating are projected to the detector plane (*z* = 0.3 m) with ∼ 40 μm of vertical blur. Under monochromatic illumination, the blur would not occur.

### 5.1. Shearing

**Figure 8:**
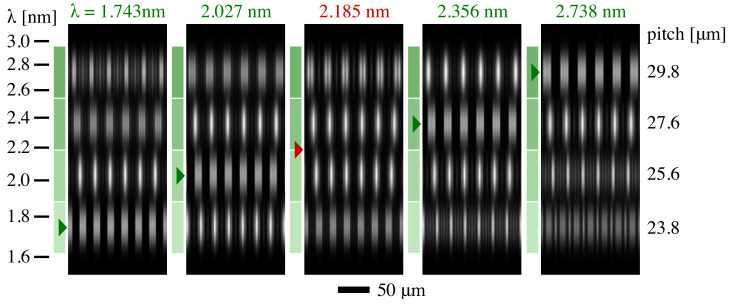
Modeled shearing detector-plane intensity patterns. The four gratings of Column 2 are illuminated with wavelengths shown above each detail. Grating pitch values are shown on the right. Square-wave intensity patterns are reproduced at the four design wavelengths (green markers), while the intermediate wavelength (red marker) shows that gratings from the two middle rows can both be used. The green colored rectangles align to the scale at the left and show the wavelength ranges over which each grating is designed to be used.

The projected light intensity was calculated for shearing measurements from the four gratings in Column 2 of the array. Central intensity details are shown in Figure 8 for five illumination wavelengths: four tuned to the grating designs and one intermediate value. The grating’s square-wave pattern is reproduced in the measured intensity, at the design wavelengths. For the intermediate wavelength, both of the central gratings can be used in wavefront analysis, although the dynamic range of measurement with either grating individually will be reduced, depending on the analysis method used.

### 5.2. Hartmann

**Figure 9:**
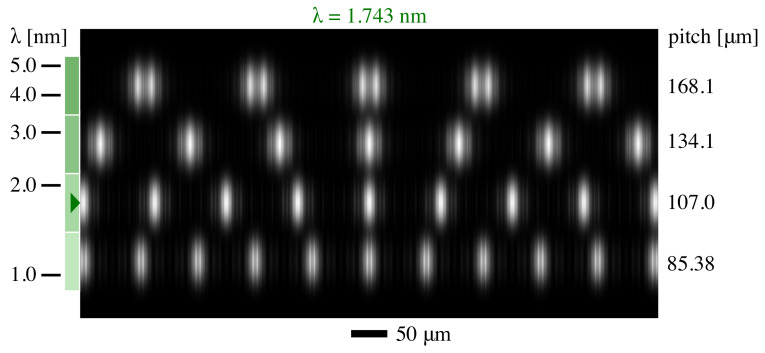
Hartmann detector-plane intensity pattern detail, calculated for the wavelength corresponding to the design of the second-lowest row (green marker). The beamlets in that row have a smooth intensity profile and minimal overlap. Green colored rectangles align to the scale at the left and show wavelength ranges over which each grid is designed to be used. We observe that the two grids designed for larger wavelengths project spots with a more prominent structure, while grids designed for smaller wavelengths show adjacent beamlet interference.

The four Hartmann grids of Column 4 were illuminated under the same conditions as above. Figure 9 shows a measured intensity calculation made for the design wavelength of the second lowest row. The fine fringes seen in the bottom row come from interference among neighboring beamlets. In the top two rows, line widths designed for larger wavelengths lead to higher Fresnel numbers.

### 5.3. Modeling Aberrated Wavefronts

**Figure 10:**
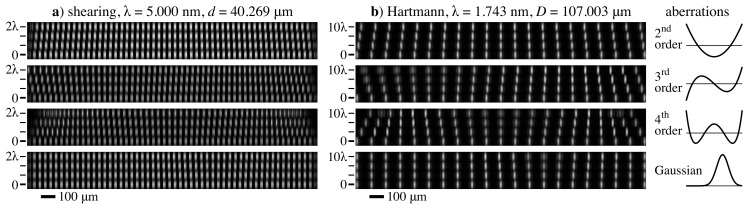
(**a**) Shearing and (**b**) Hartmann aberration modeling showing four different aberration types and varying amplitudes. Each row is a detail extracted from an intensity calculation performed for a single grating or grid on a 2 mm wide domain. The scale bars on the left show the uniformly-weighted aberration rms amplitudes for the polynomials and the peak height for the Gaussian cases.

In both shearing and Hartmann measurements, the projected intensity patterns reveal the slope of the illuminating wavefront, including its cylindrical component and aberrations. Calculations were performed for one shearing grating and one Hartmann grid, illuminated at their design wavelengths (Figure 10). Within the figure, each row represents a 2 mm wide intensity detail corresponding to the top grating of Column 3 (shearing) or the second-lowest grid of Column 4 (Hartmann).

Four types of horizontal-direction aberrations are modeled: orthonormal second-, third-, and fourth-order polynomials, and an off-center Gaussian bump. Within each group, the phase error varies from zero (unaberrated, reference case) to two waves root-mean-square (rms) for shearing and zero to 10 waves for Hartmann. For the Gaussian case, on a normalized domain from [−1,1], the wavefront path length error follows $s\left(x\right)=A\exp\{-{\left[\left(x-0.3\right)/0.3\right]}^{2}\}$. Fifty-three grating lines are illuminated in the shearing cases, and some edge effects from the finite grating are apparent. Fringes are displaced relative to the reference case, and intensity changes are visible with the largest aberrations. For both shearing and Hartmann, the local wavefront curvature controls the effective distance parameter, $z^{\prime }$. For shearing, this affects the fringe visibility. For Hartmann, the Fresnel number changes and modifies the beamlet shape, but the centroid position we seek to measure will be preserved. In this way, the range of measurable aberrations will depend on the local curvature, the wavelength, the distance, and the analysis methods being used. Hibino studied the measurement dynamic range of single-grating shearing [34].

## 6. Discussion

The wavefront sensor described here for use in vertically-dispersed and/or high-powered, narrow X-ray beams performs one-dimensional wavefront measurements as feedback for an adaptive X-ray optic. The patterned grating mirror, in an off-plane, conical diffraction mounting, is intended to be inserted and withdrawn from the beam for intermittent measurements. It deflects the beam onto a scintillator and microscope, mounted off-axis so as not to obstruct the original beam path.

Fixing the detector distance simplifies the mechanics: an array of wavelength-specific gratings enables operation across a wide energy range. With several gratings illuminated at one time, the grating most closely matching the Talbot condition (for shearing) or the Hartmann test conditions is selected for analysis. Each individual pattern spans a range of operating energies.

If the design allows, multiple grids of the same pitch can be placed with a lateral offset to enable analysis with phase-shifting approaches or to increase the density of measurement points. The combination of shearing and phase shifting has benefits for noise reduction [3]. In practice, we note that pitch values should be designed to account for the varying distance from each grating or grid to the detection plane, in the glancing-incidence geometry.

The top surface finish is important to preserve, so high-quality silicon wafers or X-ray mirror substrates are options. Silicon wafers used in our prototype had an rms height error of 0.7 nm across a 100 μm by 50 μm area, after processing. Thin-film surface coatings, such as metal films or multilayers, could be applied to increase the angular operating range or improve the X-ray reflectivity.

Curvature (figure error) in the top surface is a concern that is mitigated, to a large extent, by the small size of the individual gratings. If residual curvature is present, it imparts a static systematic error that can be removed. For this reason, it is useful to have a means to reference the lateral pattern position from one measurement to the next. Since alignment marks or features placed close to the patterns could interfere with measurement, we recommend strategies such as mutually aligning the bright center line of each grid or grating within a column.

The deep pattern etching and the layout of horizontal walls can support two-dimensional patterns for two-dimensional wavefront sensing as well, provided the patterned mirror is long enough to intercept the full beam, and sources of blur (e.g., from dispersion or a large source size) are minimized. With two-dimensional measurements, the surface figure specifications become more strict.

Tender X-ray and hard X-ray applications require thick walls to block the reflections from the etched regions; extreme ultraviolet and soft X-ray devices can use walls below 50 μm thick.

Owing to the relatively large scale of the pattern features, low-cost photolithography can be used in the device fabrication; the narrowest line in the presented design is 8.8 μm. As described in Goldberg et al. [18], the required etch depth depends on the separation between the horizontal walls and the angle of incidence. For a 2° incidence, with 50 μm walls separated by 1.458 mm, the minimum etch depth is 25.5 μm: depth >(Ltanθ)/2.

To achieve the highest possible accuracy, distortion in the scintillator-microscope system must be well characterized and calibrated in the analysis where necessary. Given the small fields of view and low magnification ratios, commercially available microscope objectives may meet the requirements for image quality with low distortion.

As mentioned above, phase-shifting, patterned mirrors (i.e., a surface relief structure) could be used for shearing interferometry, using nanometer-scale etch depths and the incidence angle to modulate the path length difference. However, creating a device to operate across a broad energy range would be challenging. Furthermore, since the Hartmann approach requires the high contrast that the binary amplitude reflection pattern provides, a chip designed for both techniques should be amplitude modulating.

The contrast and fine features in measured shearing interferograms are affected by the illumination coherence. Marathe has demonstrated using this dependence as a probe of transverse coherence [35].

In sum, we believe that this approach represents a practical, new approach for wavefront sensing on high-flux beamline optical systems—particularly in the soft X-ray and tender X-ray energy range. Rapid insertion and removal of the patterned chip will provide intermittent wavefront testing to support dynamic alignment of adaptive elements. While single-grating shearing and Hartmann testing are now well known and widely used, this work addresses several challenges: reflection geometry to mitigate thermal issues (for shearing), measurement with narrow beam widths, compatibility of shearing and Hartmann in a single element for increased wavefront measurement range and sensitivity, compact arrays of gratings to span a broad operating energy range, and simplified mechanical systems (no *z* translation required). The geometric design decisions described here are tailored to our requirements. Optimal designs for wide beams or narrow energy ranges may find different values. We are now engaged in prototyping a complete system for testing.
